# Delayed postoperative bleeding following lobectomy caused by the sharp tip of a suture: a case report

**DOI:** 10.1186/s40792-020-01039-w

**Published:** 2020-10-06

**Authors:** Tomoyuki Kawamura, Hisashi Suzuki, Moriyuki Kiyoshima

**Affiliations:** Department of Thoracic Surgery, Ibaraki Prefectural Central Hospital, 6528 Koibuchi, Kasama, Ibaraki 309-1793 Japan

**Keywords:** Postoperative bleeding, Haemothorax, Lobectomy

## Abstract

**Background:**

Postoperative bleeding is a rare but serious complication occasionally caused by hard surgical materials, such as staples. Postoperative hemorrhage caused by sutures is very rare.

**Case presentation:**

A 75-year-old man with lung cancer underwent right lower lobectomy. Eleven days after surgery, he developed a haemothorax. Emergency thoracotomy revealed arterial bleeding from a pinhole injury in the parietal pleura caused by a monofilament non-absorbable suture tip used during the initial surgery.

**Conclusions:**

Postoperative bleeding is a serious complication, and as sutures are often used in surgery, it is important to be cautious while using this material.

## Background

Postoperative bleeding is a rare, but serious complication. Previous studies have reported that bleeding occurred after pulmonary lobectomy in 2.1% of cases, and surgical materials, such as staples, can be the cause [1, 2]. However, a postoperative hemorrhage caused by sutures is rare. Herein, we report an unusual case of delayed postoperative bleeding caused by a commonly used suture.

## Case presentation

**Fig. 1:**
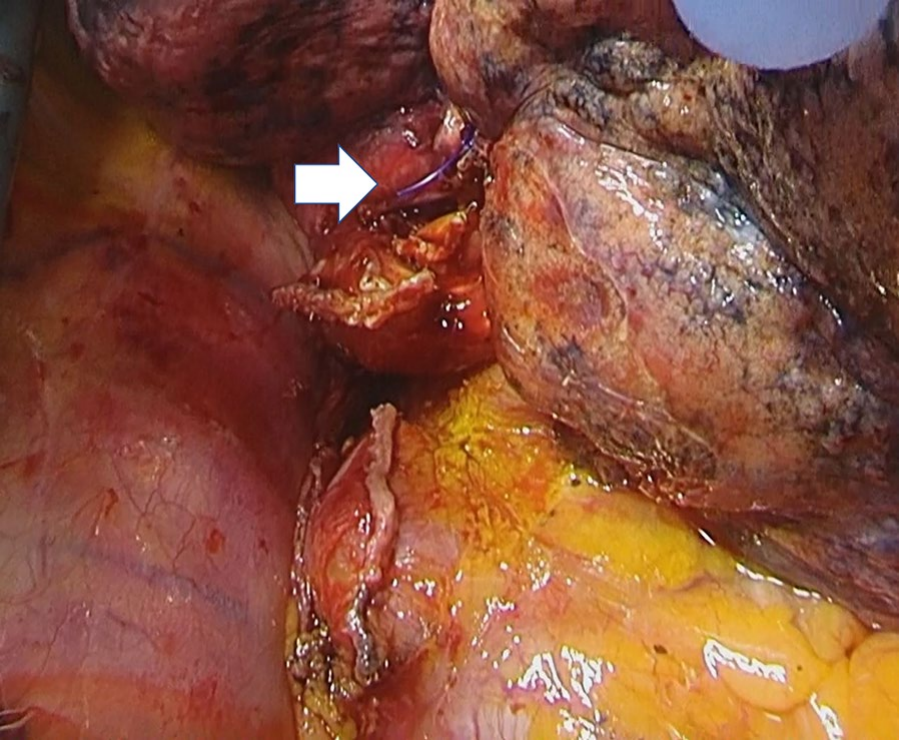
A surgical view of the initial operation showing that the Prolene® suture cut to 1 cm (arrow) that was used to close the pleural defect

**Fig. 2:**
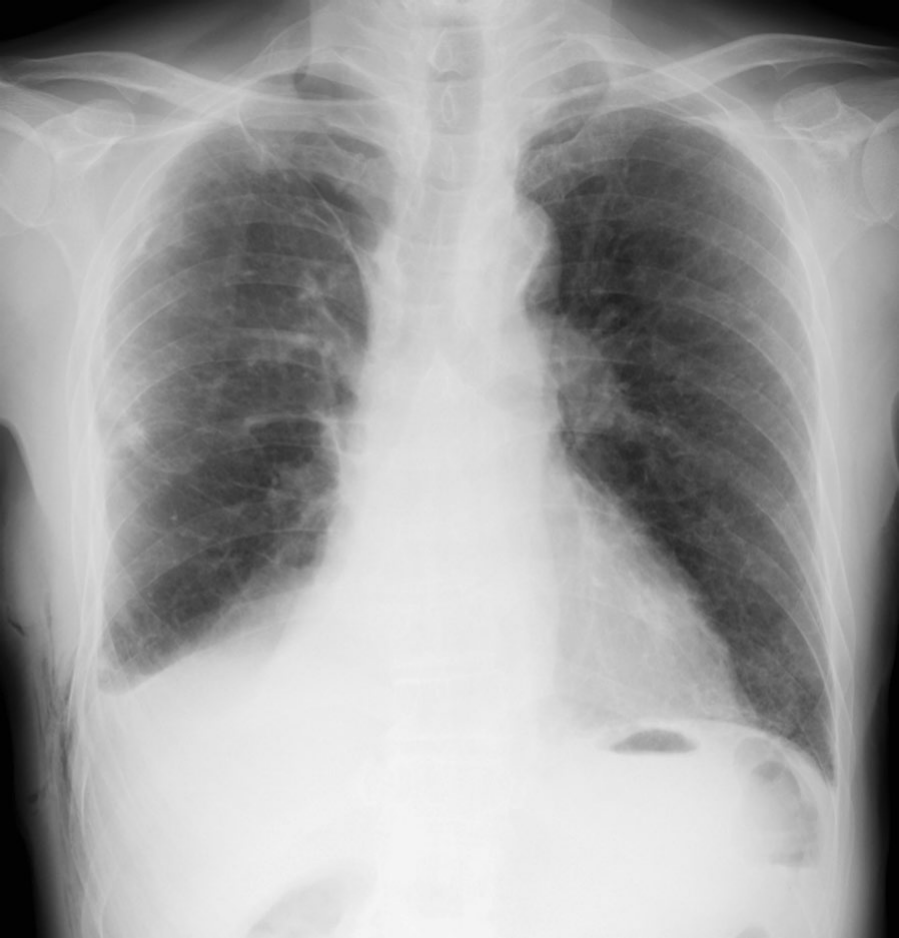
Chest X-ray of the patient before he was discharged from the initial hospitalization showing no problems after right lower lobectomy

A 75-year-old man was admitted for clinical T3N1M0 Stage IIIA primary lung cancer and underwent a right lower lobectomy by thoracotomy. He had paroxysmal atrial fibrillation and was taking an anticoagulant for it, which was discontinued before surgery. The operation went smoothly, but an air leak from the middle lobe was observed at an interlobar pleural defect, where adhesion to the lower lobe was detached. Lung tissue around the pleural defect was thick and hard because of inflammation and fibrosis. Therefore, the defect was closed using 2–0 polypropylene sutures (Prolene®, Ethicon, Bridgewater, NJ, USA). The suture was cut to 1 cm (Fig. 1). The postoperative course was uneventful, and intake of the anticoagulant was resumed 3 days after surgery. The patient was discharged 6 days after surgery, and no abnormal signs were confirmed on the chest X-ray at the day of discharge (Fig. 2).

**Fig. 3:**
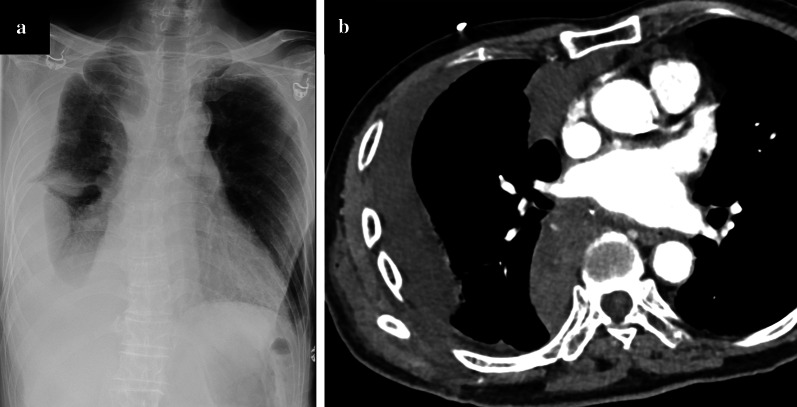
**a** Chest X-ray and **b** contrast-enhanced chest computed tomography of the patient showing a right haemothorax (performed in the emergency room)

However, on postoperative day 11, he developed dyspnoea and was transported to our emergency room. His systolic blood pressure and heart rate were 73 mmHg and 68 beats/minute, respectively. Chest X-ray and contrast-enhanced chest computed tomography (CT) revealed a haemothorax (Fig. 3). Although we could not identify extravasation of the contrast medium on the CT images, the patient was diagnosed with postoperative bleeding and was transferred to an operation room for emergency thoracotomy.

**Fig. 4:**
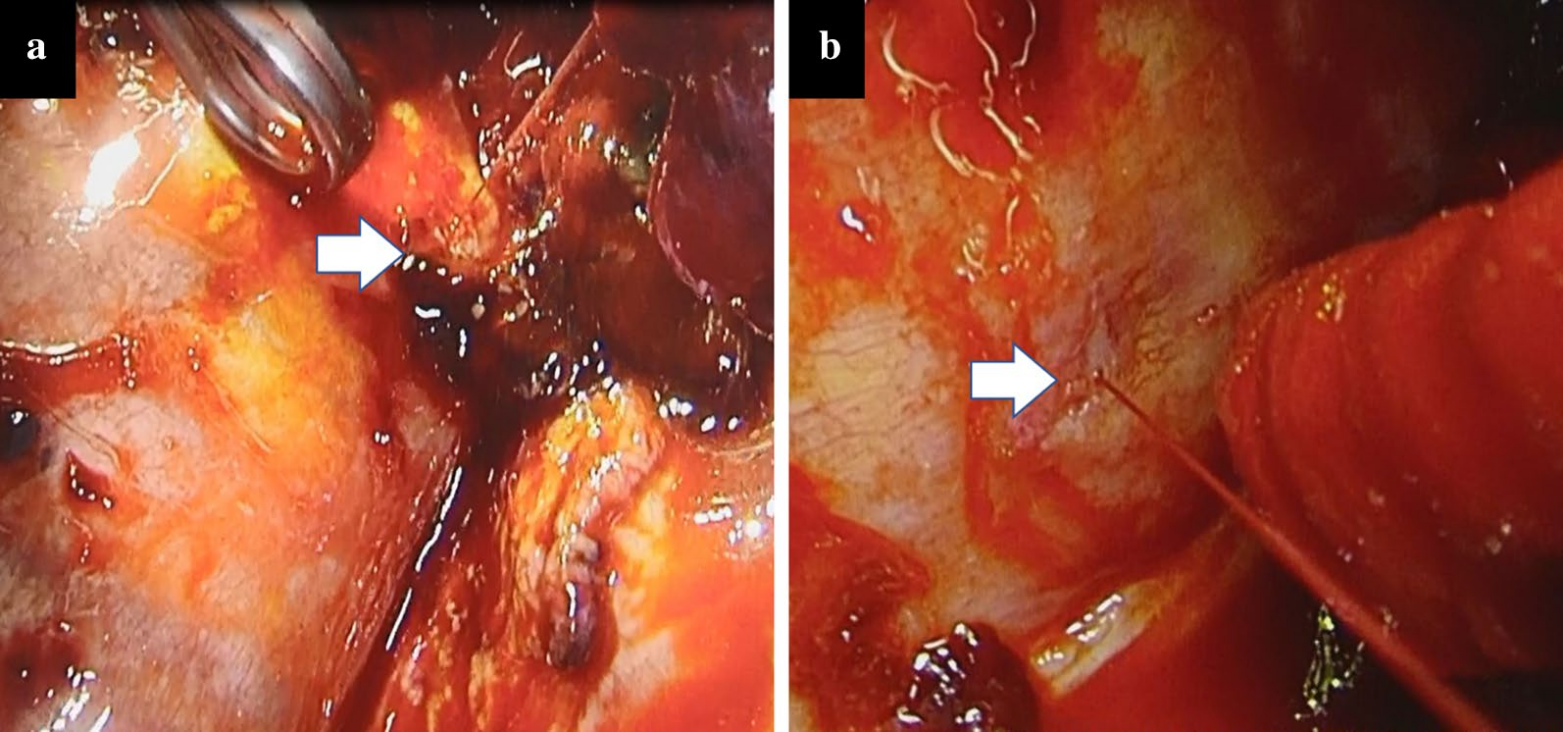
**a** A surgical view and **b** close-up view of the second operation showing active arterial bleeding from a pinhole injury (arrow) found on the anterior side of the thoracic vertebra

The intrathoracic cavity contained a large amount of clotted blood. The total amount of the clot and blood collected from the thoracic cavity was about 2000 g. After the clotted blood was removed, we discovered arterial bleeding from a pinhole in the parietal pleura anterior to the thoracic vertebra (Fig. 4) and identified an injured intercostal artery as the source. The pinhole injury was sutured and covered with a fibrin sealant patch (TachoSil®, Takeda Austria GmbH, Linz, Austria). After haemostasis, we detected a suture tip from the initial surgery protruding from the lung. When the lung inflated, the suture tip hits the bleeding point; therefore, we identified that the suture caused damage to the pleura and the artery. Careful observation revealed no other bleeding sites, and the patient was discharged 10 days later.

## Discussion

Postoperative bleeding after lung resection is a rare complication, but in cases where it occurs, the chest wall is the most commonly reported bleeding site [1, 2]. Typically, bleeding stems from injury to intercostal arteries and can be caused by surgical staples [2]. However, postoperative bleeding can result from other materials, as well. Otsuka et al. [3] described a haemothorax caused by a polydioxanone suture which was in contact with the chest wall. Similarly, bleeding in the present case was caused by a 2–0 polypropylene suture tip which was in contact with an intercostal artery.

The present case differed from other cases in terms of the delayed bleeding onset. Most cases of postoperative hemorrhage occur within 24 h of surgery and delayed bleeding is rare [2, 4]. Early bleeding caused by staples in contact with the chest wall has often occurred following partial resection of the lung [2, 4, 5]. We considered that the difference in the bleeding onset in the present case was due to the proportion of the lung that was resected. Following partial resection, a larger residual lung volume remains compared to the residual lung volume that remains after lobectomy. Therefore, lung expansion to fill the intrathoracic cavity after partial resection happens sooner than in case of lobectomy, and early contact between surgical materials and the pleura occurs, resulting in immediate bleeding [4]. In the present case, we performed lobectomy to treat lung cancer. The extra time taken for the residual lung volume to fill the intrathoracic cavity following surgery was presumed to delay the contact between the suture tip and the intercostal artery, which was the main reason for delayed bleeding. It is necessary to recognize that severe postoperative bleeding can occur not only in an early phase after surgery, but also in a later stage with delayed onset of bleeding.

The 2–0 suture was chosen to close the pleural defect in this case, because the tissue to be sutured was very thick due to inflammation and fibrosis. However, if the surrounding tissue is not thick enough to be sutured, thinner threads should be chosen. The thinner sutures bend easily and help preventing undesired damage. In fact, in another case report showing postoperative bleeding due to a suture tip, the thread size was also 2–0 [3]. Furthermore, injury may have occurred, because the suture was cut to 1 cm. This short length prevented the thread from bending when it was vertically in contact with the parietal pleura. Therefore, the sutures should be short enough to avoid contact and should also be cut to prevent a sharp edge [4]. Regarding the material of the thread, we used non-absorbable sutures to stop the air leak. We do not believe that the material of the thread was a contributing factor in this case, but it might be better to consider the use of absorbable sutures instead of non-absorbable sutures.

Alternatively, bleeding could be avoided by placing interpositions between the sharp suture tip and the parietal pleura, such as a polyglycolic acid sheet or an oxidized regenerated cellulose mesh.

## Conclusion

Sutures are frequently used during surgery, and though sutures are not as hard as staples, they can cause postoperative bleeding. Therefore, surgeons should note that sutures can be dangerous and must be handled with care. Furthermore, precautions such as cutting suture threads to appropriate lengths, reducing sharp edges, and covering tips with surgical sheets should be taken to avoid injury.

## Data Availability

Not applicable.

## Abbreviation

CT: Computed tomography
